# Prevalence, Predictors, and Reasons for Permanent Tooth Extraction Among High School Students in Saudi Arabia: A National Cross-Sectional Study

**DOI:** 10.7759/cureus.28687

**Published:** 2022-09-02

**Authors:** Ammar Almarghlani

**Affiliations:** 1 Department of Periodontics, Dental College, King Abdulaziz University, Jeddah, SAU

**Keywords:** reasons, school children, pediatric dentistry, public health, tooth loss

## Abstract

Background

Tooth loss is a major dental health concern that has adverse consequences on the remaining dentition and on the patient’s general well-being. This present study aimed to assess predictors and causes of permanent tooth extraction among students.

Methods

This national cross-sectional study in Saudi Arabia included a random sample of school students of both genders from grades 10 to 12 (15-18 years of age) and spanned the period of September 2012 to January 2016. Demographic, social, and medical history were recorded. Moreover, a list of possible reasons for tooth extraction was discussed with participants and their parents. The questionnaire was divided into two parts. They first asked for the patient's gender, age, marital status, education level, history of smoking, and the time of the last dental visit. Periodontal and dental examinations were performed. Multivariable logistic regression was used to determine predictors of tooth loss among the sample.

Results

A total of 2,435 school students were included in the study. Notably, 24% of the students had extractions of at least one permanent tooth. Nearly 27% of female students had a permanent tooth extraction compared with only 21.7% of male students, which was statistically significant. Students who visited dentists regularly had significantly more tooth extractions (39%) than students who did not (20.6%). Multivariate logistic regression analysis showed that the significant predictors for permanent tooth extraction were age, regular dental visits, and mean probing depth (PD). Caries (15%) followed by orthodontic treatment (6%) were the main reason for permanent tooth extraction among the sample.

Conclusion

Caries was responsible for most of the tooth loss among the study population. Significant predictors for permanent tooth extraction were age, regular dental visits, and mean probing depth. It follows that there is a need for intensified oral health education and awareness programs in the population with an emphasis on the prevention of dental caries.

## Introduction

Tooth loss is a major dental health concern that has adverse consequences on the remaining dentition and on the patient’s general well-being [1]. It could result from caries and its sequelae, periodontal disease, trauma, infection, or malignancies [2]. This may differ, however, from one patient to another depending on socioeconomic and educational levels [3]. Failure to replace a missing tooth may affect balance in the stomatognathic system and trigger chains of adverse effects [4]. These may include masticatory problems and chewing difficulties, altered speech, loss of self-confidence, concern about appearance, and feelings of bereavement [5-7]. Tooth loss may also lead to rotation and drifting of adjacent teeth and supra-eruption of opposing teeth, which may, in turn, result in facial asymmetry or bite collapse. A tooth loss prevention strategy is essential for esthetic, occlusal functions, socio-economical, and psychological reasons. Dental and periodontal diseases are prime examples of epidemic and widespread health problems in many nations, especially in developing countries [8]. This can be prevented and treated if diagnosed early. In such countries, restoring the natural tooth may be costly, and extractions may be an inexpensive alternative in terms of finances and time [9]. Caries is the main cause of tooth loss in developed countries such as Canada, Finland, New Zealand, Scotland, France, Norway, Sri Lanka, Sweden, and Malaysia. Periodontal diseases, including periodontitis and gingivitis (and caries, which have been reported to be high in the Saudi population, and in developing countries, such as India), are the main cause of dental extractions [10,11]. However, caries and periodontal disease seem to cause almost equal percentages of tooth loss in another group of countries, for example, the United States [12]. Patterns of oral disease and management are changing. A shift in emphasis from extraction to prevention (as of the incidence of dental caries) is decreasing, thereby bringing about the aim of preserving as much tooth structure as possible. This shift may have an influence on reasons for tooth extraction. Despite improvements in different prevention and operative modalities, tooth extraction remains an important treatment option [13]. Tooth loss may be also due to trauma or prosthodontic causes. Another reason for tooth extraction is orthodontic treatment. For instance, Ng’ang’a reported that orthodontic treatment accounted for 13% of extractions in the Nairobi population [14]. Research to identify reasons for tooth extraction has been carried out in many countries and mainly in adult populations [15]. There have been no recent studies on the prevalence of tooth loss and reasons for tooth extraction in school students in Saudi Arabia. While prior research indicates that dental caries remains by far the greatest contributor to tooth loss [9], followed by periodontal disease [10], the role of socioeconomic, age, gender, and other health characteristics is less clear.

The present study aimed to assess the prevalence, predictors, and reasons for permanent tooth extraction among school students and to synthesize data on the association between various predictors and tooth extraction in order to predict the future outcome of tooth loss based on the relation to the studied variables.

## Materials and methods

Study design

This study was a descriptive cross-sectional study performed to assess the predictors and reasons for permanent tooth extraction among school students in Saudi Arabia.

Ethical considerations

The study was approved by the Research Ethics Committee of the Faculty of Dentistry, King Abdulaziz University (073-09-12).

Inclusion and exclusion criteria

The study included and aimed at sampling a randomly selected group of healthy school students and a group of school students with medical conditions that did not have a correlation with periodontitis. The students were from grades 10 to 12 (15-18 years old) and were of both genders. Students of parents who refused to sign the consent form or who rejected the periodontal examination and students with medical conditions (conditions reported to have a relation to periodontitis) were excluded from the study. No students were admitted to the study without their parent's approval.

Research tool

A random sample of school students from grades 10 to 12 (15-18 years of age) of both genders was included. The study spanned from September 2012 to January 2016. A “Multistage Clustered Sampling Design” was employed and was reported in detail in an earlier report [11]. Subject name, gender, age, address, and socioeconomic status were recorded. At the time of examination, the examiners reviewed the medical and dental histories of the students and confirmed the said history with the parents. They then recorded the information. Dental history included the history and causes of permanent tooth extraction. Periodontal and dental examinations were performed on permanent teeth only. Primary teeth (if present), partially erupted teeth, and third molars were excluded from the examination. The gingival and periodontal examination consisted of measurement of the gingival and periodontal supporting tissue, including gingivitis, attachment loss, and probing pocket depth (using the Williams probe). We randomly selected one maxillary and one mandibular quadrant using simple random sampling. The disease was evaluated at the mesiobuccal, mid-buccal, and distolingual (MB-B-DL) of all teeth, excluding third molars following the partial mouth 3 protocol. There was also an assessment of tooth mobility, gingival bleeding, dental plaque, dental calculus, and gingival recession. For oral hygiene evaluation, we used the Silness and Loe plaque index [8]. This index is used in many epidemiological studies. It provides a quantitative assessment of plaque and concerns the thickness of plaque along the gingival margin. To visualize the plaque, teeth were dried with air, the plaque was not stained, and the plaque score was recorded from 0-3 according to the following criteria:

0 = No plaque

1 = Thin film of plaque at the gingival margin, visible only when scraped with an explorer

2 = Moderate amount of plaque along the gingival margin; interdental space free of plaque; plaque visible with the naked eye

3 = Heavy plaque accumulation at the gingival margin; interdental space filled with plaque

The severity of gingival inflammation was assessed using the Loe & Silness Gingival Index according to the following criteria:

0 = Normal gingiva, no inflammation, no discoloration, no bleeding

1 = Mild inflammation, slight color change, mild alteration of gingival surface, no bleeding

2 = Moderate inflammation, erythema, swelling, bleeding on probing or when pressure applied

3 = Severe inflammation, severe erythema and swelling, tendency toward spontaneous hemorrhage, some ulceration

Statistical methodology

This study was analyzed using IBM SPSS™ 22.0.0 (©SPSS Inc., USA). This is a simple descriptive statistic used to define the characteristics of the study sample variables through the form of counts and percentages for categorical and nominal variables, while mean and standard deviation are used to present the continuous variables. To test the bivariate relationship between tooth loss and categorical and continuous variables, this study used the chi-square and independent t-test, respectively. These tests were done with the assumption of normal distribution. Multivariable logistic regression was used to determine predictors of tooth loss among the study sample. A model with backward conditional elimination with the enter criterion=0.05 and elimination=0.10 was utilized. Statistical significance was set at P <.05.

## Results

The total number of students in the selected cities were Riyadh (n=713), Jeddah (n=657), Dammam (n=506), Abha (n=297), and Tabuk (n=262). Table 1 shows the bivariable association between the study samples’ characteristics and previous permanent teeth extracted. It shows that 24% of the students had an extraction of at least one permanent tooth. Almost 27% of the female students had a permanent tooth extraction compared to only 21.7% of male students, which was statistically significant. Nearly 28% of smokers had a history of tooth loss while only 23% of non-smokers lost permanent teeth. This was not statically significant. Students who visited dentists regularly had significantly more tooth extractions (39%) than students who did not (20.6%). In terms of brushing frequency, the more students brush their teeth, the more extraction of permanent teeth was found. Students with a history of periodontal disease showed significantly more extracted permanent teeth than students with healthy periodontium (30% and 23%, respectively). Percentages of clinical attachment loss (CAL) ≥1 mm have a significant relationship with previous permanent teeth extracted.

**Table 1:** Characteristics of the 2435 study samples relative to previous permanent teeth extracted a-significant using the chi-square test @ <0.05 level; b-significant using the independent t-test @ <0.05 level; c-significant using Welch's t-test @ <0.05 level; Note: Numbers do not add up in some cells due to missing data PD: probing depth; CAL: clinical attachment loss

Variables		Previous Permanent Teeth Extracted		P-value	
		Yes	No		
Total N (%)		585 (24.0%)	1850 (76.0%)	N/A	
Age Mean ± SD		17.38 ± 1.0	17.22 ± 1.0	0.02^b^	
Nationality N (%)	Other nationalities	49 (25.0%)	147 (75.0%)	0.73	
	Saudi	536 (23.9%)	1703 (76.1%)		
Gender N (%)	Male	288 (21.7%)	1041(78.35%)	0.03^a^	
	Female	297 (26.9%)	809 (73.1%)		
Smoker N (%)	Yes	56 (27.9%)	145 (72.1%)	0.18	
	No	529 (23.7%)	1705 (76.3%)		
Regular Dental Visits N (%)	Yes	178 (39.1%)	277 (60.9%)	<0.01^a^	
	No	407 (20.6%)	1573 (79.4%)		
Brushing N (%)	Yes	530 (24.6%)	1626 (75.4%)	0.73	
	No	55 (19.7%)	224 (80.3%)		
Brushing Frequency N (%)	Once	200 (22.8%)	678 (77.2%)	0.04^a^	
	Twice	218 (23.6%)	704 (76.4%)		
	More than 2 times	112 (31.5%)	244 (68.5%)		
Flossing N (%)	Yes	67 (26.3%)	188 (73.7%)	0.37	
	No	518 (23.8%)	1662 (76.2%)		
Tongue Brushing N (%)	Yes	216 (24.8%)	655 (75.2%)	0.50	
	No	369 (23.6%)	1195 (76.4%)		
Previous Dental Treatment N (%)	Yes	540 (32.6%)	1117 (67.4%)	<0.01^a^	
	No	45 (5.8%)	733 (94.2%)		
Periodontal Disease N (%)	No	522 (23.5%)	1704 (76.5%)	0.03^a^	
	Yes	63 (30.1%)	146 (69.9%)		
Missing Teeth Mean ± SD		1.05 ± 1.4	0.20 ± 0.7	<0.01^c^	
Mean PD Mean ± SD		0.65 ± 0.2	0.57 ± 0.2	<0.01^c^	
Mean CAL Mean ± SD		0.07 ± 0.2	0.04 ± 0.1	0.02^c^	
PD (%) ≥4 mm Mean ± SD		2.16 ± 5.2	1.76 ± 4.7	0.10	
CAL (%) ≥1 m Mean ± SD		3.53 ± 13.2	2.22 ± 10.3	0.03^c^	

Table 2 shows the results of the multivariable logistic regression models. Controlling for other variables, significant predictors for permanent tooth extraction were age, regular dental visits, and mean pocket depth (PD). Increased age, more regular dental visits, and increased PD were associated with more tooth extraction.

**Table 2:** Binary logistic regression model of the characteristics of the 2435 study samples relative to previous permanent teeth extracted b-significant using the binary logistic regression model, with backward conditional elimination with the entry criterion=0.05, elimination=0.10, model fit measures. -2 log-likelihoods: 2271.325, AIC: 2285.325, R2 (Cox & Snell): 0.066, R2 (Nagelkerke): 0.099; c-No is the reference PD: probing depth

Variables in the Equation	OR	95% C.I. for EXP(OR)		P-value
		Lower	Upper	
Age	1.2	1.3	1.0	0.016^b^
Smoker (Yes vs No) ^c^	1.5	2.2	1.0	0.054
Regular Dental Visits (Yes vs No) ^c^	1.4	2.0	1.17	0.002^b^
Tongue Brushing (Yes vs No)^ c^	0.78	1.0	0.60	0.053
Gingival Index	1.16	1.3	1.0	0.086
Mean PD	3.84	8.33	1.72	0.001^b^
Constant	687.090			<0.001^b^

Table 3 shows the causes of tooth extraction among the students. It shows that the majority of the sample (n=2435) had extractions of a permanent tooth due to caries (15% of the total sample) followed by orthodontic treatment (6% of the total sample). Only 1% of the students had extractions of a permanent tooth due to periodontal disease (mobility) and another 1% due to trauma.

**Table 3:** Reasons for the history of permanent tooth extraction among the study sample

Previous Permanent Teeth Extracted		
Reason for tooth extraction	Yes	No
Caries N (%)	354 (15%)	2018 (85%)
Orthodontic treatment N (%)	151 (6%)	2284 (94%)
Mobility N (%)	34 (1%)	2401 (99%)
Trauma N (%)	21 (1%)	2414 (99%)

## Discussion

Tooth loss can provide information regarding the availability of dental care, the prevalence of dental disease, and attitudes toward tooth loss. In the present study, 24% of the students had extractions of at least one permanent tooth. It was then found that 40.9% in a cross-sectional study carried out in the Eastern Province of Saudi Arabia on the same age group also had such extractions [11,16].

Despite numerous previous studies having examined the nature of tooth loss and dental extractions of permanent teeth in adults, surprisingly little information exists regarding the loss of permanent teeth among students [17]. One must be cautious in interpreting the comparison of the studies’ results due to cultural and demographic differences and the availability of dental services in different countries. When comparing the results of independent studies carried out at different points of time, care must be taken since various sources of bias may invalidate the comparisons [18].

The present study shows a tendency for more extraction of permanent teeth among females than males. This is consistent with some earlier studies [19-21]. Other studies, however, showed a greater percentage of permanent tooth extraction among males than females [12,22,23]. It should be noted that these studies sampled different age groups than the present study. The increased teeth extractions among females could be due to more frequent dental visits for the management of inadequate and unaesthetic dentition than they being a true reflection of their higher prevalence of tooth mortality than males [7,24]. It is important to note that attending regular dental visits is a very important protective factor against periodontal disease. Since the present study was conducted on children (who have a lower prevalence of destructive periodontal disease than adults), it was shown that students who visited dentists regularly and brushed their teeth more frequently had significantly more extractions of permanent teeth than students who did not. In addition, some of these extractions may be due to financial issues, as restoring teeth is more expensive and time-consuming than extracting them [25]. Another reason contributing to these results could be the need for permanent tooth extraction because of therapeutic reasons (i.e., orthodontic treatment), which was the second most common reason for tooth extraction among the students. Nevertheless, the importance of attending regular dental visits must be emphasized to allow for the early detection and treatment of emerging oral diseases, including periodontal conditions, and for the evaluation and reinforcement of oral hygiene measures.

Regarding the extraction of permanent teeth, several conclusions were obvious from the data. First, caries was the most common reason for extraction. These results are consistent with a study conducted in the US, which reviewed 2000 records and concluded that 53% of tooth extraction was mainly due to caries and pulpal pathosis [17]. Another study reported that caries accounted for 81% of tooth extraction in UAE [26].

In the present study, tooth extraction due to periodontitis was found in only 1% of the sample. This finding is consistent with a study on an Italian sample that reported that no one under 20 years of age had extractions due to periodontal diseases [27]. These findings contradict a study that included Sudanese subjects aged above 17 years, which reported that periodontitis accounted for 34.8% of tooth loss. The difference in the presented data could be due to cultural differences or variations in the socioeconomic status and accessibility to dental care between the samples [28].

The prevalence of malalignment (mainly crowding) and the need for orthodontic treatment among the Saudi population were reported to be high [29]. Therefore, not surprisingly, tooth extraction for orthodontic reasons was the second main reason for permanent tooth extraction in the study sample [30]. These results are consistent with another study that included a Saudi sample [20].

As limitations to the current study that can be included in future studies, patient compliance should be studied in association with the reason for tooth extraction. In addition, detailed periodontal bacterial pathogenic screening tests, including a Porphyromonas gingivalis species count, as well as saliva buffering capacity and production tests can be explored in order to test the degree of association with the reasons for tooth extraction.

## Conclusions

The present study indicated that caries is the principal reason for tooth extraction among the studied population of school children in Saudi Arabia, followed by extraction for orthodontic reasons. Therefore, prevention of caries in the population must begin early in life to identify those at risk. Certainly, this study and many others support the need for further studies defining these risk factors and exploring methods of intervention for those at risk.
